# Resilience and life quality of health professionals in capital of Turkey during COVID-19 pandemic: Özge Tonbuloğlu Altıner

**DOI:** 10.1093/eurpub/ckac131.076

**Published:** 2022-10-25

**Authors:** Ö Tonbuloğlu Altıner, A Uğraş Dikmen, C Gingir, S Özkan

**Affiliations:** Public Health, Gazi University, Ankara, Turkey

## Abstract

**Background:**

COVID-19 pandemic has had significant effects on physical and mental health of health professionals. It is thought that resilience protects individuals against mental illness and helps individuals cope with difficulties and stress more effectively. In this study, it was aimed to evaluate resilience, life quality and related factors of health professionals during COVID-19 pandemic.

**Methods:**

A cross-sectional study was performed among health professionals working at a tertiary hospital in Turkey’s capital Ankara. An occupation based stratified sampling was done with taking alpha 0.05 and 1-beta 0.80. A questionnaire that consists of sociodemographic information, COVID-19 Impact on Quality of Life Scale and Connor Davidson Resilience Scale was used to collect data. The results of scales were divided into two parts by taking the median values as cut off points. Descriptive/inferential statistics and logistic regression were performed on IBM’s SPSS 27.0 program.

**Results:**

A total of 987 participants were surveyed. 66% of them were female, and the average age was 36. Multivariate logistic regression analysis results that physicians (OR:1.48, 95% CI:1.05-2.07, p = 0.024) and nurses (OR:1.46, 95% CI:1.08-1.97, p = 0.013) have lower resilience. The impact of COVID-19 on quality of life was higher for the following groups; physicians (OR:2.07, 95% CI:1.43-3, p < 0.001), nurses (OR:1.61, 95% CI:1.10-2.36, p = 0.013), who have bachelor/higher degrees (OR: 1.54,95% CI: 1.02-2.31,p=0.038), infected with COVID-19 (OR:1.33, 95% CI:1.02-1.74, p = 0.034), have COVID-19 related relative lost (OR:1.42, 95% CI:1.06-1.89, p = 0.016), and live with risk groups (OR:1.31, 95% CI:1.01-1.71, p = 0.042).

**Conclusions:**

Physicians and nurses who take care of patients one-on-one have lower resilience and higher decrease in life quality due to COVID-19 impacts. This result indicates a significant quality drop in health services is inevitable during pandemics and should be considered by the policy makers.

**Key messages:**

• Policies should be developed to increase the resilience of healthcare professionals so that they can effectively combat public health emergencies such as COVID-19 and not affect their quality of life.

• It is necessary to determine risk groups among health workers and plan training programs to increase resilience.

